# Calcitonin gene-related peptide regulates periodontal tissue regeneration

**DOI:** 10.1038/s41598-024-52029-z

**Published:** 2024-01-16

**Authors:** Koji Miki, Noboru Takeshita, Motozo Yamashita, Masahiro Kitamura, Shinya Murakami

**Affiliations:** Department of Periodontology and Regenerative Dentistry, Osaka University Graduate School of Dentistry, 1-8 Yamadaoka, Suita, Osaka 565-0871 Japan

**Keywords:** Neuroscience, Stem cells, Anatomy, Diseases

## Abstract

Calcitonin gene-related peptide (CGRP), a neuropeptide composed of 37 amino acids secreted from the sensory nerve endings, reportedly possesses various physiological effects, such as vasodilation and neurotransmission. Recently, there have been increasing reports of the involvement of CGRP in bone metabolism; however, its specific role in the pathogenesis of periodontitis, particularly in the repair and healing processes, remains to be elucidated. Therefore, this study aimed to investigate dynamic expression patterns of CGRP during the destruction and regeneration processes of periodontal tissues in a mouse model of experimental periodontitis. We also explored the effects of CGRP on periodontal ligament cells, which can differentiate to hard tissue-forming cells (cementoblasts or osteoblasts). Our findings demonstrated that CGRP stimulation promotes the differentiation of periodontal ligament cells into hard tissue-forming cells. Experimental results using a ligature-induced periodontitis mouse model also suggested fluctuations in CGRP expression during periodontal tissue healing, underscoring the vital role of CGRP signaling in alveolar bone recovery. The study results highlight the important role of nerves in the periodontal ligament not only in sensory reception in the periphery, as previously known, but also in periodontal tissue homeostasis and tissue repair processes.

## Introduction

The periodontal ligament, a collagen-rich non-calcified connective tissue, is situated between the cementum and alveolar bone. Its primary role is to provide support and secure teeth via a connective tissue attachment. When subjected to mechanical forces such as occlusion and orthodontic pressures, the periodontal ligament maintains a width of 100–250 μm. This triggers remodeling in the alveolar bone, cementum, and connective tissue, crucial for preserving periodontal tissue homeostasis^1^. Recent studies have identified a subset of cells within the periodontal ligament that retain characteristics of mesenchymal stem cells, displaying pluripotency. Periodontal ligament cells can differentiate into either cementum-forming cells (cementoblasts) or bone-forming cells (osteoblasts). These cells play a pivotal role in wound healing and periodontal tissue regeneration^2–4^. Furthermore, the periodontal ligament receives abundant sensory innervation as a sensory receptor during occlusion. However, with aging, the periodontal ligament shows narrowing of its space, reduction in cellular components, and degeneration of nerve fibers^5–7^_._ This suggests that nerve-related molecules are intricately involved in the pathogenesis of age-related periodontal diseases and systemic conditions, as well as in the maintenance of periodontal tissue homeostasis and wound healing processes.

Neurons transmit stimuli received by sensory organs, which are relayed to the central nervous system, and central commands to the peripheral nervous system as electrical signals; however, they also produce and secrete neuropeptides, which regulate the activities of surrounding tissues. Previous histological searches have revealed that the periodontal ligament contains Ruffini nerve endings as mechanoreceptors and free nerve endings as nociceptors^8^. Furthermore, neuropeptides have been found to be present in large numbers in free nerve endings. Among the various neuropeptides present in periodontal tissues^9^, one neuropeptide reportedly related to bone metabolism is the calcitonin gene-related peptide (CGRP), which is produced by selective splicing of the calcitonin gene and is composed of 37 amino acids. CGRP is abundant in perivascular nerves^10,11^ and is a potent vasodilator; its synthesis is presumably enhanced in tissues undergoing inflammatory reactions^12^. Neuropeptides in periodontal tissues are produced in the trigeminal ganglion and secreted from peripheral nerve endings. Interestingly, nerve fibers containing CGRP are reportedly abundant in the periodontal ligament^13,14^, and experimental tooth movement affects nerve fibers containing CGRP^15,16^.

The molecular and cellular mechanisms of CGRP in bone metabolism have gradually become clearer. Recent studies have demonstrated that fracture healing is impaired in bones with damaged nerves distributed on the periosteum, whereas high CGRP secretion promotes surrounding bone formation^17^. Moreover, impaired fracture healing has been reported in CGRP-deficient mice^18^.

In light of this background, it is plausible that nerves within the periodontal ligament also release CGRP within the same tissue, exerting specific physiological effects on cementum and alveolar bone metabolism. However, limited studies have explored CGRP-dependent bone metabolism within periodontal tissues.

Therefore, this study aimed to investigate the function of CGRP in periodontal ligament cells, which play a main role in periodontal tissue regeneration. Furthermore, we examined the function of CGRP in a ligature-induced periodontitis mouse model, aiming to dissect the role of CGRP in the wound healing processes of periodontal tissues.

## Results

### Expression of CGRP receptor in mouse periodontal tissue and MPDL22 cells

**Figure 1:**
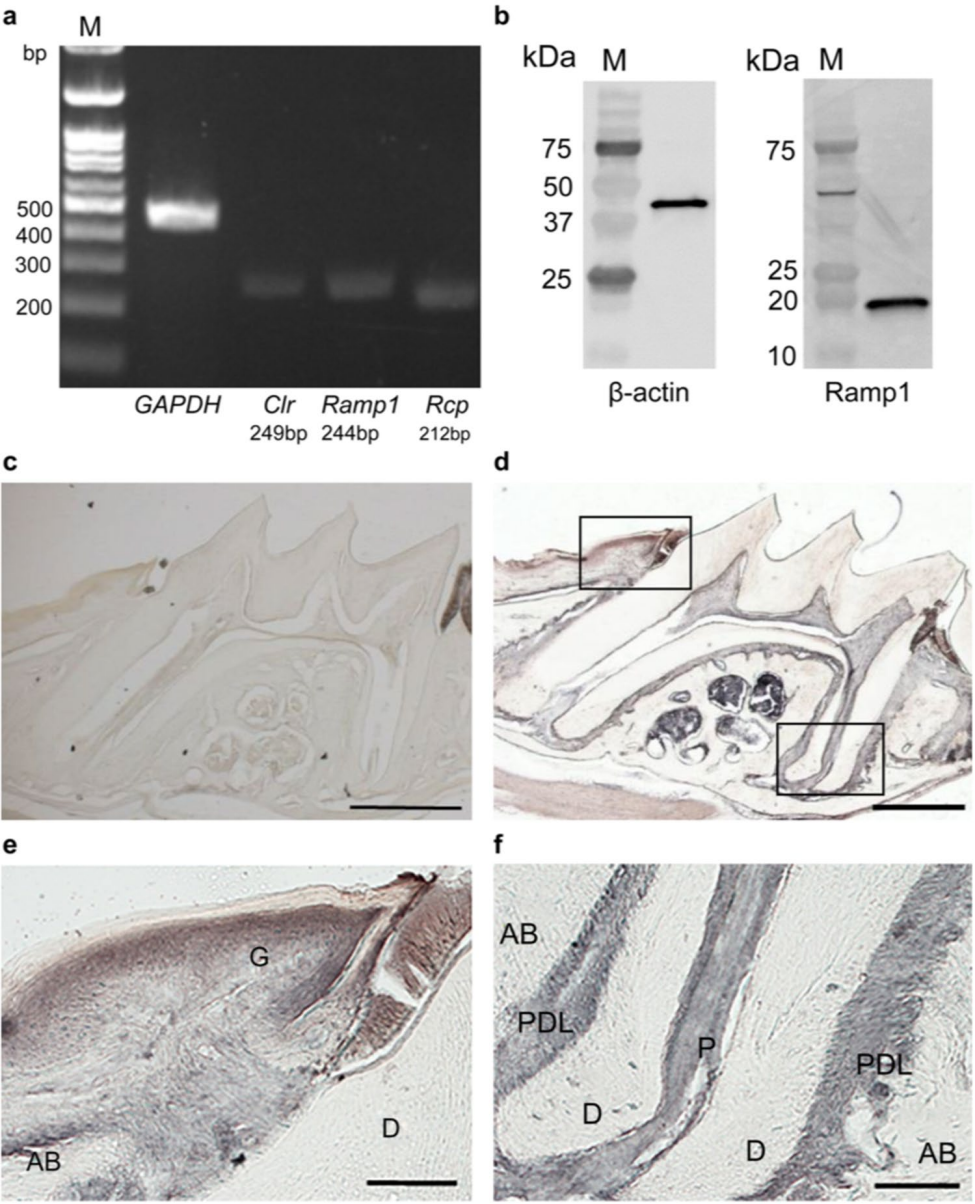
Expression of calcitonin gene-related peptide (CGRP) receptor in mouse periodontal tissue and MPDL22 cells. (**a**) Receptor activating modifying protein 1 (*Ramp1*), calcitonin receptor-like receptor (*Clr*), and receptor component protein (*Rcp*) expression in MPDL22 cells was analyzed using reverse transcription-polymerase chain reaction. Gene expression was observed in all the components. (**b**) A whole cell fraction of MPDL22 cells was collected and subjected to western blotting with a specific antibody for RAMP1. RAMP1-positive reaction was observed. Original blots/gels are presented in Supplementary Fig. 1 (a, b1, b2). (**c**) Immunostaining image of a mouse maxillary first molar without primary antibody (control). (**d**) Immunostaining image of a mouse maxillary first molar with anti-RAMP1 antibody. (**e**) Enlarged image of the upper left square in D. RAMP1-positive reaction was observed in the entire gingiva except the stratum corneum. (**f**) Enlarged image of the lower right square of D. RAMP1-positive reaction was observed in the pulp and periodontal ligament. *AB* alveolar bone, *D* dentin, *G* gingiva, *P* pulp, *PDL* periodontal ligament. Scale bar: 500 μm (**c**,**d**) 100 μm (**e**,**f**).

We examined CGRP receptor expression in mouse periodontal tissue and MPDL22 cells, a periodontal ligament cell line. mRNA expression of CGRP receptor components in MPDL22 cells was examined by reverse transcription-PCR. The results demonstrated that *Ramp1*, *Clr*, and *Rcp* mRNA were expressed in MPDL22 cells (Fig. 1a). Furthermore, we examined the receptor activity-modifying protein 1 (RAMP1) expression in the MPDL22 cells at the protein level by western blotting. The results clearly revealed a signal band at 14–18 kDa (Fig. 1b). In addition, immunohistochemical staining of the tissue sections of maxillary molars from BALB/c mice with anti-RAMP1 antibody revealed RAMP1 expression in the gingiva, pulp, and periodontal ligament, excluding the stratum corneum (Fig. 1c–f).

### Effect of CGRP during induction of differentiation of MPDL22 cells into hard tissue-forming cells

**Figure 2:**
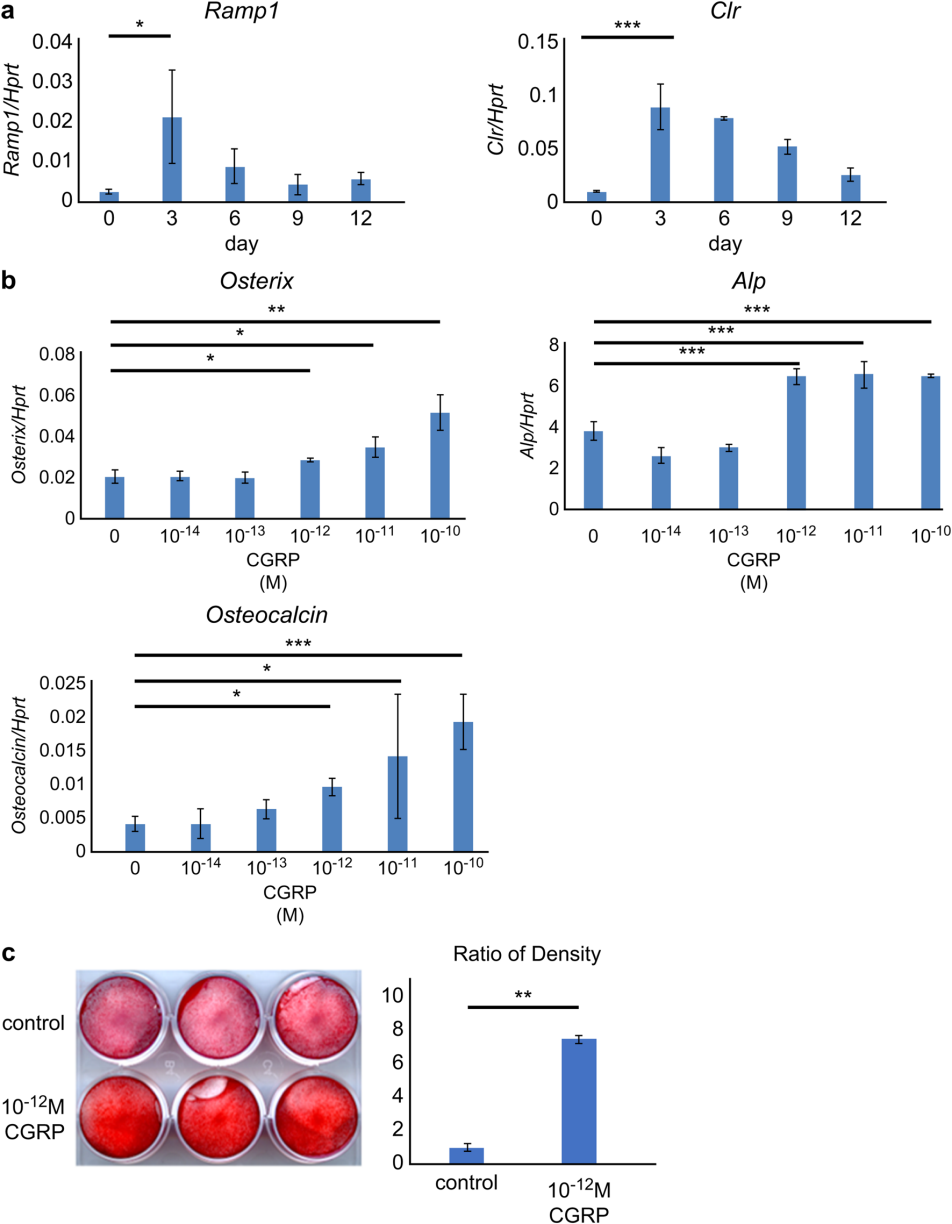
Effect of calcitonin gene-related peptide (CGRP) on the process of induction of cytodifferentiation of MPDL22 cells into hard tissue-forming cells. (**a**) Real-time polymerase chain reaction of *Ramp1* and *Clr* during the cytodifferentiation process in the absence of CGRP. On day 3, the expression of CGRP receptor was significantly increased. (**b**) Induced cytodifferentiation in the presence of 10^−14^ M, 10^−13^ M, 10^−12^ M, 10^−11^ M, and 10^−10^ M CGRP, Real-time PCR was performed for *Osterix* on day 3, *Alp* on day 6, and *Osteocalcin* on day 12. CGRP stimulation caused calcification in MPDL22 cells. CGRP stimulation significantly upregulated the expression of calcification-related genes (*Osterix, Alp,* and *Osteocalcin*) in MPDL22 cells. (**c**) Alizarin staining image on day 18 after induction of cytodifferentiation CGRP stimulation significantly promoted the formation of calcified nodules. Statistical significance was determined by Tukey’s test (**a**,**b**) and Student's-*t* test (**c**). *P < 0.05, **P < 0.01, ***P < 0.001.

We examined the kinetics of CGRP receptor expression during differentiation of MPDL22 cells: real-time PCR was performed for *Ramp1* and *Clr* expression. The results demonstrated a significant increase in *Ramp1* and *Clr* expression on day 3 of differentiation induction (Fig. 2a), followed by a decrease in their expression. Next, MPDL22 cells differentiation was induced in the presence of 10^−14^ M, 10^−13^ M, 10^−12^ M, 10^−11^ M, and 10^−10^ M CGRP, and total RNA extraction and cDNA production were performed at days 0, 3, 6, 9, and 12 of differentiation induction. The calcification-related gene expression was subsequently analyzed by real-time PCR, and the expression of *Osterix* on day 3, *Alp* on day 6, and *Osteocalcin* on day 12 was significantly increased by culturing in the presence of 10^−12^ M CGRP or higher (Fig. 2b). Furthermore, differentiation was induced in the presence of 10^−12^ M CGRP until day 18, and calcification nodules were analyzed by alizarin staining, which demonstrated a significant increase in staining in the 10^−12^ M CGRP-stimulated group compared with that in the control group (Fig. 2c).

### Examination of the dynamics of CGRP-positive nerves in a ligature-induced periodontitis model in mice

**Figure 3:**
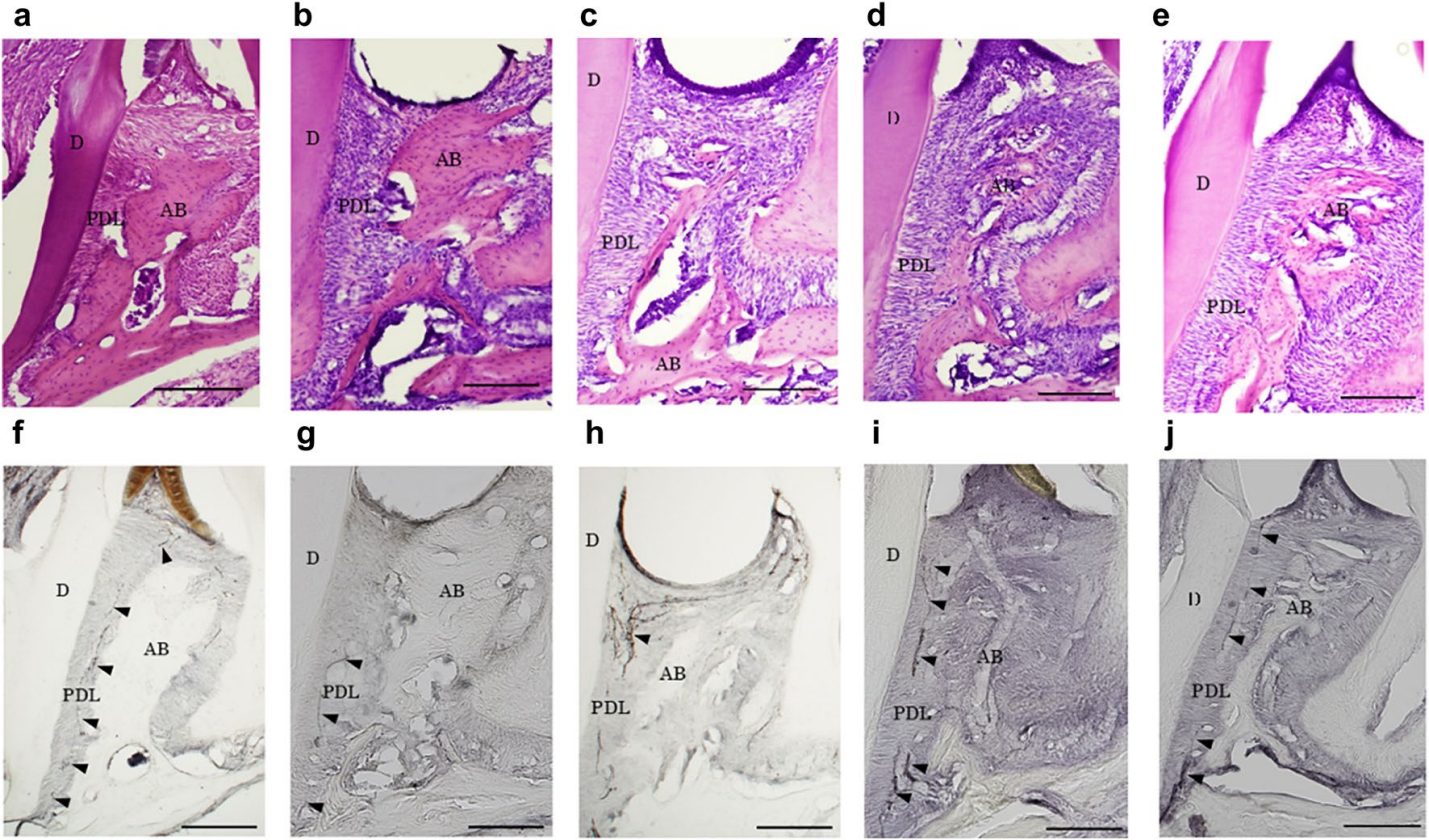
Examination of the dynamics of CGRP-positive nerves in a ligature-induced periodontitis model in mice. A total of 15 mice were prepared, and 5-0 silk threads were ligated on the maxillary bilateral second molars of 12 of them. Three mice without silk thread ligation were euthanized, in whom μCT imaging and tissue collection were performed on day 0 of the experiment. After silk thread ligation, three mice were euthanized on days 3 and 7, and μCT imaging and tissue collection were performed in the same manner. The remaining six mice had the silk threads removed on day 7, and μCT imaging and tissue collection were performed on day 10 (day 3 of silk thread removal) and day 14 (day 7 of silk thread removal) of the experiment. Sections were then prepared from the obtained tissues, Hematoxylin and eosin (HE) staining was performed to determine cell and tissue structure, and immunohistochemical staining with anti-CGRP antibody was performed to examine the dynamics of CGRP-positive nerves. HE staining images of mouse silk ligation periodontitis model on days 0, 3, 7, 10, and 14 (**a**–**e**), and immunostained images with anti-calcitonin gene-related peptide (CGRP) antibodies on days 0, 3, 7, 10 and 14 in the mouse silk ligation periodontitis model (**f**–**j**). (**a**) HE-stained image on day 0. (**b**) HE-stained image on day 3. (**c**) HE-stained image on day 7. (**d**) HE-stained image on day 10. (**e**) HE-stained image on day 14. Progressive bone destruction was observed from day 0 to day 7 during silk ligation, while bone tissue repair was observed from day 7 to day 14 after silk removal. In addition, a strong inflammatory cell infiltrate was observed in the periodontal ligament tissue on days 3–10; however, this infiltrate was mild on day 14. (**f**) Immunostaining image with anti-CGRP antibodies on day 0. CGRP-positive nerves were evenly distributed throughout the periodontal ligament tissue. (**g**) Immunostaining image with anti-CGRP antibody on day 3. CGRP-positive nerves disappeared in the periodontal ligament immediately below the ligature. (**h**) Immunostaining image with anti-CGRP antibody on day 7. CGRP-positive nerves increased and accumulated in the periodontal ligament immediately below the ligature. (**i**) Immunostaining image with anti-CGRP antibody on day 10. The increase and accumulation of CGRP-positive nerves have ceased. (**j**) Immunostaining image with anti-CGRP antibodies on day 14. CGRP-positive nerves returned to an even distribution in the periodontal ligament tissue. Arrowheads indicate both CGRP-positive nerves. *AB* alveolar bone, *D* dentin, *PDL* periodontal ligament, scale bar: 100 µm (**a**–**e**).

Based on the above observations, we investigated the function of CGRP in the periodontium in vivo using a mouse model of silk ligation periodontitis. The HE-stained images demonstrated progressive bone destruction on days 0–7 during silk thread ligation and bone tissue repair on days 7–14 after silk thread removal (Fig. 3a–e). In our models, a strong inflammatory cell infiltrate was observed in the periodontal ligament tissue on days 3–10 (Fig. 3b–d); however, by day 14, the observed infiltrate was mild (Fig. 3e).

Immunostaining images revealed that CGRP-positive nerves before ligation were approximately evenly distributed throughout the periodontal ligament (Fig. 3f). On day 3 after ligation, CGRP-positive nerves were lost in the periodontal ligament immediately below the ligature site (Fig. 3g). On day 7 after ligation, CGRP-positive nerves increased and accumulated in the periodontal ligament just below the ligature (Fig. 3h). On day 10 after ligation (3 days after silk thread removal), the expression of CGRP-positive nerves decreased compared with that on day 7 (Fig. 3i). On day 14 after ligation (7 days after silk thread removal), the CGRP-positive nerves had almost returned to their original distribution (Fig. 3j).

### Alveolar bone destruction and healing in a ligature-induced periodontitis model using Ramp1^−/−^ mice

**Figure 4:**
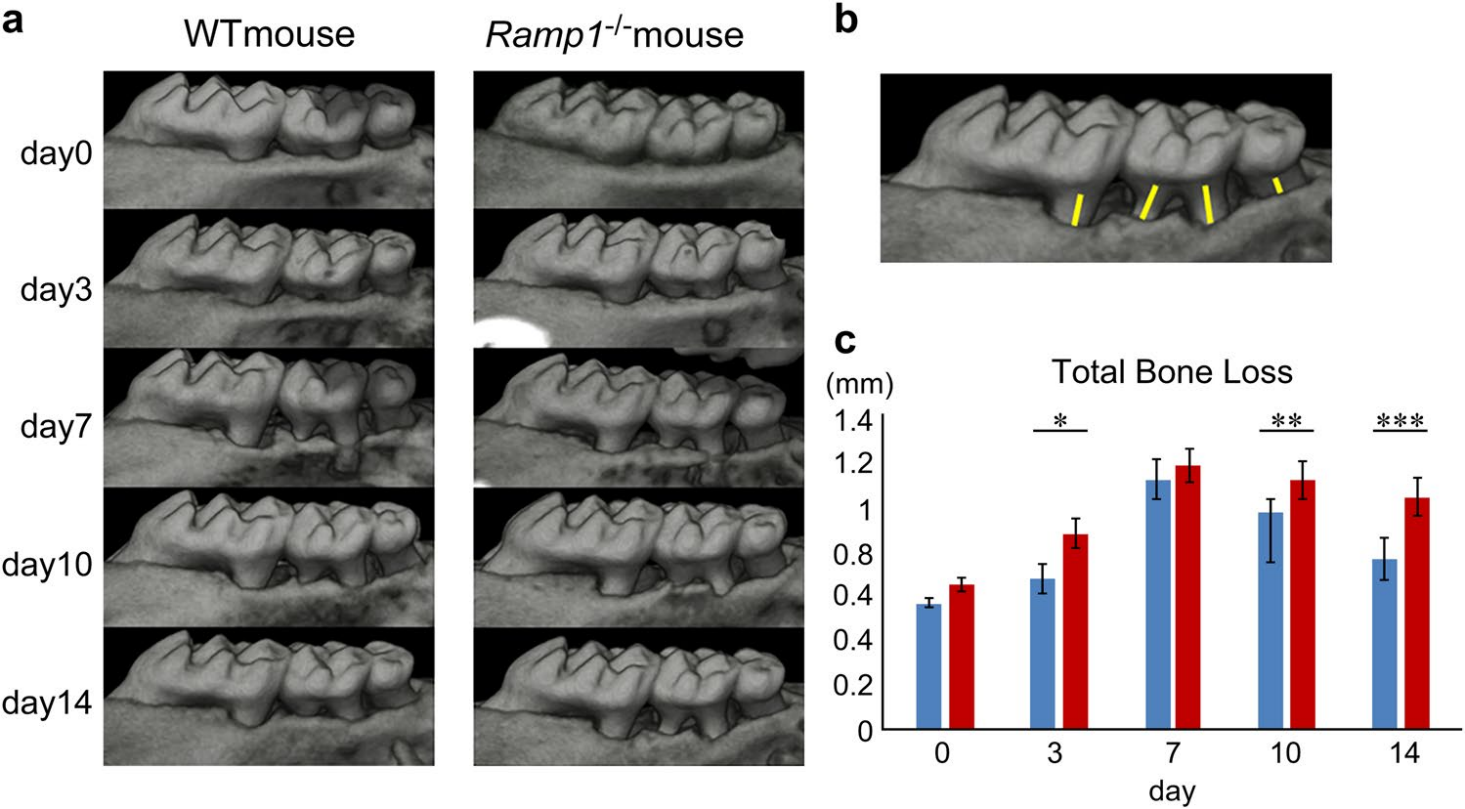
Investigation of periodontal tissue destruction and healing in wild-type and *Ramp1*^−/−^ mice using micro computed tomography (μCT) imaging. (**a**) μCT images of wild-type and *Ramp1*^*−/−*^ mice on ligation days 0, 3, 7, 10, and 14. (**b**) As indicated by the yellow line, the distance from the cemento-enamel junction to the alveolar crest was measured for four roots: the distal root of the maxillary first molar, the mesial and distal roots of the maxillary second molar, and the mesial root of the maxillary third molar, and the total was evaluated as the Total Bone Loss. (**c**) Total Bone Loss of wild-type and *Ramp1*^−/−^ mice at days 0–14 is shown in the graph Statistical significance was determined by Tukey’s test (**c**) (blue: wild-type mice, red: *Ramp1*^−/−^ mice, n = 5, *P < 0.05, **P < 0.01, ***P < 0.001). No significant differences in alveolar bone status between the wild-type and *Ramp1*^−/−^ mice were observed on day 0. *Ramp1*^−/−^ mice showed delayed alveolar bone recovery after silk thread removal compared with that in wild-type mice.

The above-mentioned experiments were performed in *Ramp1*^−/−^ mice in the silk thread ligation periodontitis model. The obtained μCT images (Fig. 4a) were subsequently used to compare the degradation and healing of alveolar bone between the wild-type and *Ramp1*^−/−^ mice. No significant differences were noted in the alveolar bone status between the wild-type and *Ramp1*^−/−^ mice on day 0. The significant reduction in alveolar bone recovery were noted on days 10 and 14 in *Ramp1*^−/−^ mice compared with that in wild-type mice (Fig. 4b,c).

## Discussion

In this study, our focus was to investigate the role of molecules within periodontal ligament cells, specifically their involvement in hard tissue formation within periodontal tissues, with the aim of shedding light on the function of CGRP in these tissues. In addition, we utilized a ligature-induced periodontitis mouse model to examine the pathophysiological role of CGRP, particularly in the process of alveolar bone repair.

Existing research has demonstrated that CGRP promotes bone formation in various cell types, including osteoblasts^19,20^, and bone marrow mesenchymal stem cells^20,21^, suggesting an association between CGRP and bone metabolism. However, the significance of CGRP in the function of periodontal ligament cells, which play pivotal roles in periodontal tissue regeneration, homeostasis, and wound healing, has not yet been reported. Our findings revealed that CGRP stimulation activates the differentiation process of a mouse periodontal ligament cell line, MPDL22 cells, when induced to differentiate into hard tissue-forming cells. In the present study, the RAMP1 expression in the MPDL22 cells was observed at the protein level by western blotting. However, as previously reported^22^, various recognition sites for RAMP1 antibodies are commercially available. The biological significance of the RAMP1 positive reaction in western blotting in the present study in the periodontal ligament cells is unknown. To characterize the RAMP1 expression in the periodontal ligament, further analysis is warranted. The precise mechanism of action remains to be fully elucidated, as CGRP binding to specific receptors induces various differentiation processes involving cyclic adenosine monophosphate (cAMP) and cAMP response element-binding protein (CREB), a transcription factor involved in the cell proliferation and differentiation for osteogenesis. CGRP also activates its downstream targets, *Runx2* and *SP7*^23–26^ and activates BMP2 and Wnt/β-catenin, which are regulatory pathways of CREB^27,28^. Therefore, CREB activation could promote the differentiation of periodontal ligament cells into hard tissue-forming cells by CGRP; however, further studies elucidating the mechanism are warranted.

The present study required CGRP at concentration of 10^−12^ M or higher to promote the differentiation of MPDL22 cells into hard tissue-forming cells (Fig. 2b,c). Based on the results of previous studies, the average serum concentration of CGRP was 36.3 pmol/L ± 6.2 (SD)^29^, and the concentration of 10^−12^ M of CGRP used in this experiment corresponds to a concentration that could be physiologically present in vivo. CGRP is also considered to be the most potent microvasodilator currently known^30^, exerting physiological functions at low concentrations. Injecting a femtomolar dose of CGRP increases the blood flow in the skin microcirculation and induces redness^31^. These findings suggest that even under in vitro conditions, CGRP may regulate the differentiation of periodontal ligament cells into hard tissue-forming cells. It has also been shown that the production and secretion of CGRP decrease with aging^7,32^. This could be attributed to the age-related decrease in hard tissue metabolism of periodontal ligament cells via CGRP, resulting in increased susceptibility to periodontal disease upon aging. In the future, assessing in vivo metabolism of periodontal tissue could prove essential in gaining further insights.

Our study revealed that the gene expression of CGRP receptors changes during the differentiation of periodontal ligament cells into hard tissue-forming cells. When MPDL22 cells were induced to differentiate using the calcified induction medium, *Ramp1* and Clr gene expression peaked on day 3 of induction, which is the early stage of differentiation into hard tissue-forming cells (Fig. 2a). This suggests that periodontal ligament cells could be affected by CGRP in the early stage of differentiation, thereby promoting their differentiation into osteoblasts and cementoblasts. Nagao et al*.*^33^ have previously demonstrated that β2-adrenergic receptors in the sympathetic nervous system and receptors for CGRP in the sensory nervous system, which are related to the regulation of osteogenesis, are most strongly expressed in undifferentiated osteoblasts, whereas neurokinin 1 receptors, receptors for substance P derived from sensory nerves, which are also related to osteogenesis, are not expressed in undifferentiated cells. However, its expression increases with osteoblast differentiation, peaking in mature osteoblasts. The periodontal ligament is richly innervated, and it is assumed that CGRP as well as receptors for various neurosecretory substances are expressed in the periodontal ligament cells. Neuropeptides released from peripheral nerve endings are thought to cooperate with various cytokines involved in periodontal ligament metabolism to maintain homeostasis, based on the differentiation stage of the periodontal ligament cells.

In this study, we used mice lacking the gene for RAMP1, a constitutive protein specific for the CGRP receptor^34,35^. The bone phenotypes of 8-week-old Ramp1^−/−^ mice were not particularly different from that of wild-type mice. We examined the wound healing process of periodontal tissues in a mouse model of periodontitis caused by silk thread ligation using Ramp1^−/−^ mice and found that alveolar bone repair was delayed compared with that in wild-type mice (Fig. 4c). The association of CGRP with bone metabolism in vivo has been reported in numerous studies, indicating the important role of CGRP in bone metabolism^17,18,36^. However, few reports suggest the association of CGRP in periodontal tissue repair and regeneration^37^; nevertheless, CGRP expression in the periodontal tissues reportedly changes with tissue remodeling^15,16,38,39^. This study's results suggest that CGRP signaling via RAMP1 receptors plays an important role in alveolar bone regeneration during the healing process of periodontal tissues. As one possible mechanism of action, we believe the CGRP-RAMP-1 signal may be involved in the early differentiation of periodontal ligament cells into hard tissue-forming cells. In the present study, CGRP-positive nerve fibers in the periodontal ligament immediately after silk thread ligation disappeared (Fig. 3g), but later increased and accumulated significantly as the destruction of periodontal tissue progressed. The up-regulation of CGRP expression promotes osteogenesis and typically returns to physiological expression upon repair^40^. The present study suggests that elevated CGRP expression during inflammation may promote the differentiation of tissue stem cells into hard tissue-forming cells in the periodontal ligament and the subsequent supply of osteoblasts and cementoblasts for periodontal tissue repair.

In the present study, loss of CGRP-positive nerve fibers was observed immediately after silk thread ligation (Fig. 3g); however, whether nerve fibers other than the same nerve fibers were also lost is unknown. In periodontal tissues, nerve fibers that do not contain CGRP, such as Aβ fibers with Ruffini nerve endings, are also present^8^. In the periodontal ligament, Aδ and C fibers, which are mainly nociceptors, are distributed throughout the periodontal ligament, and Ruffini nerve endings, which are mechanoreceptor Aβ fibers, are mainly expressed in the apical third of the root. Previous studies on the dynamics of periodontal ligament nerve fibers following transection of the inferior alveolar nerve in rats^41^ and on the dynamics of dental pulp nerve fibers after the formation of a tooth socket^42^ have reported temporary degeneration and loss of nerve fibers. In the present study, it is highly likely that CGRP-positive nerve fibers and peptide-free nerve fibers were simultaneously degenerated and lost due to significant tissue damage caused by silk thread ligation. Thus, the expression dynamics of CGRP-positive nerve fibers during the wound healing process in periodontal tissues is likely to reflect the expression dynamics of nerve fibers. Regarding the relationship between the expression dynamics of nerve fibers and tissue construction, peripheral nerve networks and vascular networks communicate closely during the development of peripheral tissues^43^. It is thought that nerve fibers in the healing process of periodontal tissue also presumably interact closely with the vascular network to promote healing and regeneration of the same tissue.

Many studies have demonstrated that CGRP can promote soft tissue repair and wound healing^44–46^. In addition, CGRP has been reported to promote angiogenesis^44,46^. Fibroblast growth factor (FGF)-2, the active ingredient in REGROTH^®^, a periodontal tissue regenerator, also has a potent angiogenic effect and has been shown to create a local environment suitable for periodontal tissue regeneration by establishing a lifeline of vascular networks in periodontal tissue defects^47–49^. Similarly, the angiogenic effects of CGRP could be related to the activation of periodontal tissue healing and regeneration. This is not considered in this study but is likely to be an important issue in the future.

In the future, we expect to establish a new periodontal tissue regeneration therapy by clarifying the overall function of CGRP in periodontal tissues and its mechanism and by developing a method to promote the production of CGRP intrinsically.

## Methods

### Reagents

We used recombinant mouse CGRP (Phoenix Pharmaceuticals, Burlingame, CA, USA) for the experiments.

### Cell culture

We established a mouse periodontal ligament cloned cell line, i.e., MPDL22 cells, and maintained it as described previously^50^. MPDL22 cells was grown in α-modification of Eagle's medium (Wako Pure Chemical Corporation, Osaka, Japan) supplemented with 10% fetal bovine serum (FBS) (Biowest, Nuaillé, Pays De La Loire, France) and 60 g/mL kanamycin (Wako Pure Chemical Corporation, Osaka, Japan). Eagle's medium (Wako Pure Chemical Corporation) (hereinafter mentioned as “culture medium”) with 100 ng/mL of human recombinant FGF-2 (Kaken Pharmaceutical, Tokyo, Japan) was added, and the cells were cultured under 5% CO_2_ at 37 °C and 95% humidity. For passaging, the cells were treated with phosphate-buffered saline (PBS) (Wako Pure Chemical Corporation) supplemented with 0.05% trypsin and 0.02% ethylene diamine tetraacetic acid (Thermo Fisher Scientific, Waltham, MA, USA) and seeded into 100 mm-culture dishes (Corning, Corning, NY Cells from the 37th to 40th generations of MPDL22 cells were used in the experiments.

### Induction of differentiation

To induce differentiation of MPDL22 cells into hard tissue-forming cells, the cells were seeded into 12-well cell culture plates (Corning) at 1.0 × 10^5^ cells/well and incubated in culture medium supplemented with 10 mM β-glycerophosphate (Wako Pure Chemical Corporation) and 50 μg/mL ascorbic acid (Wako Pure Chemical Corporation) (herein referred to as calcification). The culture medium was changed every 3 days thereafter.

### Total RNA extraction

Total RNA extraction was performed using the nucleic acid extraction reagent RNA-Bee™ (TEL-TEST, Friendswood, TX, USA). RNA-Bee was added to the cells collected at the end of the culture to solubilize them. After adding 1/10 volume of chloroform (Wako Pure Chemical Corporation), the cells were centrifuged at 12,000 rpm for 15 min. The separated aqueous layer was collected, and RNA was precipitated by the addition of isopropanol (Wako Pure Chemical Corporation) and washed with 75% ethanol (Wako Pure Chemical Corporation). The total RNA precipitates were then dissolved in 20-μL diethylpyrocarboxylic acid-treated water (Wako Pure Chemical Corporation, hereafter abbreviated as DEPC-treated water), and the total RNA amount was measured using a NanoDrop ND-1000 (Thermo Fisher Scientific).

### Complementary DNA (cDNA) preparation

cDNA was prepared by reverse transcription using total RNA as the template, and 1 μg of each RNA sample heat-treated at 65 °C was mixed with 20% five First Strand Buffer (Thermo Fisher Scientific), 1 mM dithiothreitol (Life Technologies, Gaithersburg, MD, USA), and 1 μg of each RNA sample heat-treated at 65 °C. The final concentration shown below was added to the sample: (Gaithersburg, MD, USA), 1.1-U/µL ribonuclease inhibitor (Takara Bio, Shiga, Japan), 0.5-mM dNTP mixture (Takara Bio), 5-U/µL Moloney-Mouse leukemia virus reverse transcriptase (Life technologies), and 55 ng/µL random Hexamers (Pharmacia Biotech, Milwaukee, WI, USA). The reaction solution was stored at 37 °C for 60 min and subsequently treated at 99 °C for 5 min to inactivate the residual enzymes for cDNA preparation.

### cDNA amplification and detection by polymerase chain reaction (PCR)

The CGRP receptor is a dimer consisting of calcitonin receptor-like receptor (CLR) and receptor activity-modifying protein 1 (RAMP1). RAMP1 is a dimer composed of CLR and RAMP1, which, together with receptor component protein (RCP), drive the intracellular signaling pathway^51^.

For the expression analysis of *Ramp1*, *Clr,* and *Rcp* in MPDL22 cells, synthesized cDNA was used as a template and amplified by PCR using primers specific for each gene. AmpliTaq Gold™ 360 Master Mix (Thermo Fisher Scientific) 12.5 μL and 1 μL of cDNA solution, and 0.2 μM of each gene-specific primer (Gene Design, Osaka, Japan and FASMAC, Kanagawa, Japan) listed in Table 1 were added at a final concentration. The reaction solution was prepared with DEPC-treated water to a total volume of 25 μL. After heat treatment of the prepared reaction solution at 95 °C for 2 min, cDNA was amplified by Mastercycler^®^nexus GX2 (Eppendorf, Eppendorf, Hamburg, Germany) for 40 cycles of thermal denaturation at 95 °C for 30 s, annealing at 56 °C for 3 s, and extension reaction at 72 °C for 1 min as a single cycle. Forty cycles of cDNA amplification were performed. These PCR products were electrophoresed on 1.5% agarose gel (Wako Pure Chemical Corporation) and visualized using ethidium bromide (Wako Pure Chemical Corporation).

**Table 1:** Primers for RT-PCR.

Gene		Primers
*Mouse Gapdh*	F	5′-TGTGTCCGTCGTGGATCTGA-3′
	R	5′-TTGCTGTTGAAGTCGCAGGAG-3′
*Mouse Ramp1*	F	5′-CTGAGACACAGCCAAGTGGA-3′
	R	5′-AAGCCAACAGCTTCAAGGAA-3′
*Mouse Clr*	F	5′-CTCCGTTTTCCTTCTGCTTG-3′
	R	5′-TCAGGAAAAAGCAAGCCACT-3′
*Mouse Rcp*	F	5′-TGCCTCTGCACCTCTGTATG-3′
	R	5′-GCACCCCTGCTCTATCTCTG-3′

*GAPDH* glyceraldehyde-3-phosphate dehydrogenase, *Ramp1* receptor activity-modifying protein 1, *Clr* calcitonin receptor-like receptor, *Rcp* receptor component protein.

### Western blotting

Whole cell fractions obtained from MPDL22 cells were used for analysis of RAMP1 and CLR protein expression, and β-actin was used as a control. The total cell fraction was reduced by heat treatment with Laemmli’s 5 × sample buffer containing 2-mercaptoethanol (Wako Pure Chemical Corporation) at 98 °C for 10 min and then incubated with Mini-PROTEAN^®^ TGX Precast Gel (Bio-Rad Laboratory) and Mini-PROTEAN^®^ Tetra System (Bio-Rad Laboratory). PROTEAN^®^ Tetra System (Bio-Rad Laboratory), and the protein lysate fraction was developed. The proteins were subsequently transferred to a membrane using Trans-Brot^®^ Turbo (Bio-Rad Laboratory) and PowerPac™ (Bio-Rad Laboratory) (room temperature, 100 V, 3 h). Membranes were blocked (room temperature, 5 min) using Bullet Blocking One for western blotting (Nacalai tesque, Kyoto, Japan) and then probed with the following primary antibodies: rabbit monoclonal anti-RAMP1 (1:1000; Abcam, Tokyo, Japan, #ab156575) and mouse monoclonal anti-β-actin (1:5000; Sigma-Aldrich, #A5316). After washing, membranes were incubated with secondary antibodies, horseradish peroxidase (HRP)-labeled goat anti-rabbit IgG antibody (1: 5000; GE Healthcare, Chicago, IL, USA) or HRP-conjugated sheep anti-mouse IgG antibody (1: 10,000; GE Healthcare) and made to react with SuperSignal West Dura Extended Duration Substrate (Thermo Fisher Scientific) to amplify the luminescence signal; bands were detected with an ImageQuant LAS 4000 (GE Healthcare). We used Precision Plus Protein Dual Color Standards (BIO-RAD, #161-0374) as a ladder.

### Real-time PCR

Real-time PCR analysis was performed using the gene-specific real-time PCR primers (Gene Design and FASMAC) listed in Table 2 with cDNA as the template (Biosystems, Foster City, CA, USA) on a Step One Plus Real-time PCR System^®^ (Applied Biosystems). The expression levels of each gene were calculated relative to the expression level of the endogenous control gene, *Hypoxanthin guanine phosphoribosyl transferase* (*Hprt*), which is one of the housekeeping genes.

**Table 2:** Primers for real-time PCR.

Gene		Primers
*Mouse Hprt*	F	5′-TTGTTGTTGGATATGCCCTTGA-3′
	R	5′-AGGCAGATGGCCACAGGACTA-3′
*Mouse Alp*	F	5′-ACACCTTGACTGTGGTTACTGCTGA-3′
	R	5′-CCTTGTAGCCAGGCCCGTTA-3′
*Mouse Osteocalcin*	F	5′-AGCAGCTTGGCCCAGACCTA-3′
	R	5′-AGCGCCGGAGTCTGTTCACTA-3′
*Mouse Osterix*	F	5′-CGCATCTGAAAGCCCACTTG-3′
	R	5′-CAGCTCGTCAGAGCGAGTGAA-3′
*Mouse Ramp1*	F	5′-GGCATTTCAGTTGGGATCAGCTA-3′
	R	5′-TGTCGCCTGCCAATGTCAGTA-3′
*Mouse Clr*	F	5′-CTCCGTTTTCCTTCTGCTTG-3′
	R	5′-TCAGGAAAAAGCAAGCCACT-3′

*Hprt* hypoxanthine guanine phosphoribosyl transferase, *Alp* alkaline phosphatase, *Ramp1* receptor activity-modifying protein 1, *Clr* calcitonin receptor-like receptor.

### Examination of calcified nodule formation ability by alizarin staining

The ability to form calcified nodules was examined by alizarin red staining. After induction of differentiation of MPDL22 cells into hard tissue-forming cells for 18 days, the culture supernatant was removed, the cell layer was washed twice with PBS, and fixed with 100% ethyl alcohol (Wako Pure Chemical Corporation) at 4 °C for 10 min. (Wako Pure Chemical Corporation) aqueous solution (pH 6.4) (room temperature, 5 min), followed by washing with distilled water. The stained images were scanned using a color image scanner GT-X970 (Epson, Tokyo, Japan). The images were then analyzed using WinRoof image analysis software (Mitani Corporation, Fukui, Japan). The fraction of stained area and color density were quantified, and the product was calculated as the degree of calcified nodule formation.

### Experimental animals

Fifteen 8-week-old male BALB/c mice and 15 *Ramp1* gene-deficient (*Ramp1*^−/−^) mice^34,35^ (weighing 17–21 g) were used for the experiments. These mice were generated, and heterozygous knockout mice were backcrossed to BALB/c mice for 12 generations. The 15 mice were divided into five groups of three mice each at 0, 3, 7, 10, and 14 days after silk ligation. They were maintained under controlled temperature (23 ± 2 °C) and light–dark cycle with free access to food and water and fed a regular chow diet (5.1% fat, 55.3% carbohydrate, 23.1% protein; MF Oriental Yeast Co., Ltd., Tokyo, Japan).

All the experiments in this study were performed in accordance with the animal experiment guide established by the Animal Experimentation Committee of the Graduate School of Dentistry, Osaka University (Animal Experimentation Committee of the Graduate School of Dentistry, Osaka University, Reception No.: Animal Dentistry R-01-019-0, Genetic Recombination Approval No.: 04484). All animal experiments complied with the ARRIVE guidelines. All efforts were made to minimize suffering.

BALB/c mice were purchased from Japan SLC Corporation (Shizuoka, Japan); Ramp1^−/−^ (BALB/c genetic background) mice were obtained from RIKEN BRC (#RBRC0285)^34,35^. On the advice of RIKEN BRC, BALB/c mice were used as experimental controls.

### Ligature-induced periodontitis model

We used a triple anaesthetic mixture of 75 µg/mL medetomidine hydrochloride (Nippon Zenyaku Kogyo, Tokyo, Japan), 400 µg/mL midazolam (Sandoz, Tokyo, Japan) and 500 µg/mL butorphanol tartrate (Meiji Seika Pharma, Tokyo, Japan). For awakening from anesthesia, an anesthetic antagonist (75 μg/mL atipamezole hydrochloride) adjusted with antisedan (Nippon Zenyaku Kogyo) and saline solution (Otsuka Pharmaceutical) was used.

Ligation and removal of silk threads were performed under deep anesthesia by intraperitoneal administration of 0.2 mL of the three anesthetic mixtures. After each procedure, the mice were awakened by intraperitoneal administration of 0.2 mL of the anesthetic antagonist. The mouse ligature-induced periodontitis model was performed according to Abe et al*.*^52^. The silk threads used were 5-0 PERMA-HAND SILK BLACK BRAIDED (Ethicon, Raritan, NJ, USA) and ligated to the maxillary bilateral second molars upon visualization under a binocular stereomicroscope.

### Micro computed tomography (μCT)

Micro tomography images of the specimens were obtained using R-mCT2 (Rigaku, Tokyo, Japan) at a tube voltage of 90 kV, tube current of 160 μA, and slice width of 5 μm. The data obtained from the imaging were processed using three-dimensional image analysis software TRI/3D-BON (RATOC System Engineering, Tokyo, Japan), and two-dimensional images were obtained by setting the Z axis so that the X and Y coordinates of the contours of the maxillary left and right molars overlapped.

The two-dimensional images were imported into the image analysis software WinRoof (Mitani Corporation). The distance from the cemento-enamel junction at the center of the root to the alveolar crest was measured for the four roots: the distal root of the maxillary first molar, the mesial and distal roots of the maxillary second molar, and the mesial root of the maxillary third molar, and the total was evaluated as Total Bone Loss (Fig. 4b).

### Preparation of the tissue sections

A catheter was inserted into the heart of 8-week-old mice that had been euthanized with CO_2_ gas. After purging with PBS, the mice were reflux-fixed in 4% paraformaldehyde phosphate buffer (Wako Pure Chemical Corporation), and the maxillary bone was removed. The maxillary bone was subsequently immersed overnight in PBS containing 20% sucrose. We prepared the frozen sections at a thickness of 20 µm from OCT blocks (Sakura Seiki, Tokyo, Japan) after they had been placed in a freezer at − 80 °C for 10 min; they were then in Leica CM3050 S (Leica, Nussloch, Germany) at − 20 °C for 10 min.

### Hematoxylin–eosin (HE) staining

HE staining was performed using Meyer hematoxylin (Mutoh Chemical, Tokyo, Japan) and 1% eosin Y solution (Mutoh Chemical). Tissue specimens were sealed in ProLong™ Glass Antifade Mountant (Thermo Fisher Scientific) and observed under an optical microscope.

### Immunostaining

The morphology of the periodontal ligament nerve fibers was observed using the ABC method. Sections were washed with PBS and treated with methanol (Wako Pure Chemical Corporation) containing 0.003% hydrogen peroxide (Wako Pure Chemical Corporation) for 30 min to inactivate endogenous peroxidase, and then washed with PBS and stained with rabbit polyclonal anti-CGRP (1:5000; Sigma-Aldrich, #C8198) and rabbit polyclonal anti-RAMP1 (1:5000; Abcam, #ab203282) and reacted for 16–18 h. Thereafter, they are washed with PBS, reacted with biotinylated swine anti-rabbit IgG (1:500; Dako, Copenhagen, Denmark) for 90 min, washed with PBS, and reacted with ABC complex (Vector Laboratories, Burlingame, CA, USA) for 90 min. After washing with PBS, ABC complex (Vector Laboratories, Burlingame, CA, USA) was reacted for 90 min. The HRP was subsequently visualized in 0.05 M Tris–HCl buffered saline, pH 7.6, containing 0.04% 3,3-diaminobenzidine (Sigma-Aldrich) and 0.003% hydrogen peroxide solution^6^ and sensitized with 0.1% nickel ammonium sulfate (Wako Pure Chemical Corporation). All the reactions were performed at room temperature. Tissue specimens were air-dried after the reactions, dehydrated in an ascending ethanol series, permeabilized with G-NOX (Genostaff, Tokyo, Japan), encapsulated in Multi Mount 480 (Matsunami Glass Industries, Osaka, Japan), and observed under an optical microscope.

### Statistical analysis

Experimental data are presented as mean ± standard error. Significant difference tests were performed using Student's-*t* test for two-group comparisons and Tukey’s test as post-hoc following analysis of variance (ANOVA) for multi-group comparisons. *P* < 0.05 was considered a significant difference.

### Supplementary Information

Supplementary Figure 1.

Supplementary Figure 2.

Supplementary Figure 3.

## Data Availability

All data supporting the findings of this study are available within the article.
