# Supplementary figures and images for: Calcitonin gene-related peptide regulates periodontal tissue regeneration
Source: Sci Rep. 2024 Jan 16;14:1344. doi: 10.1038/s41598-024-52029-z (PMC10791604; doi:10.1038/s41598-024-52029-z)

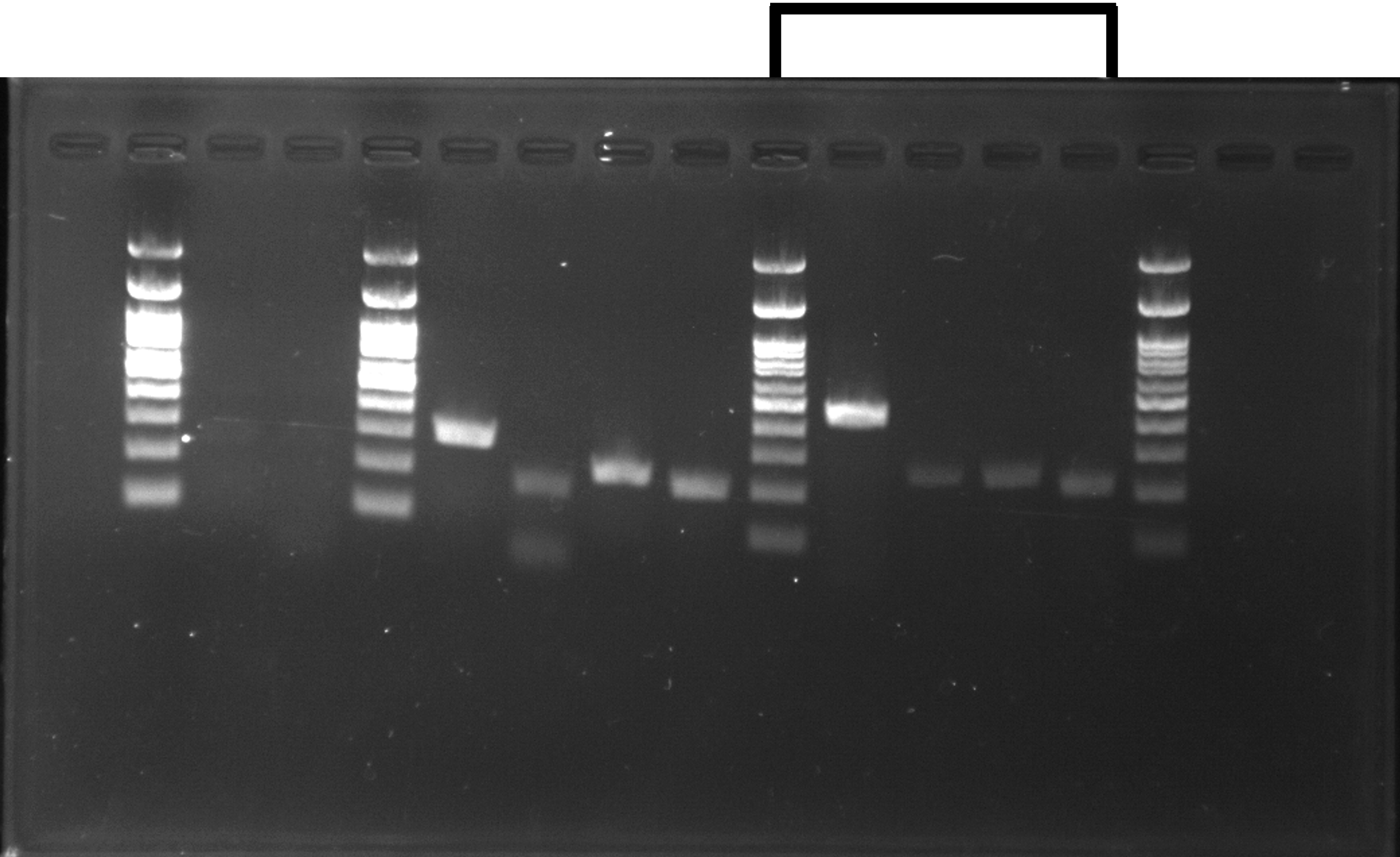

Supplement: Supplementary file 1 — Supplementary Figure 1. [file 41598_2024_52029_MOESM1_ESM.tif]

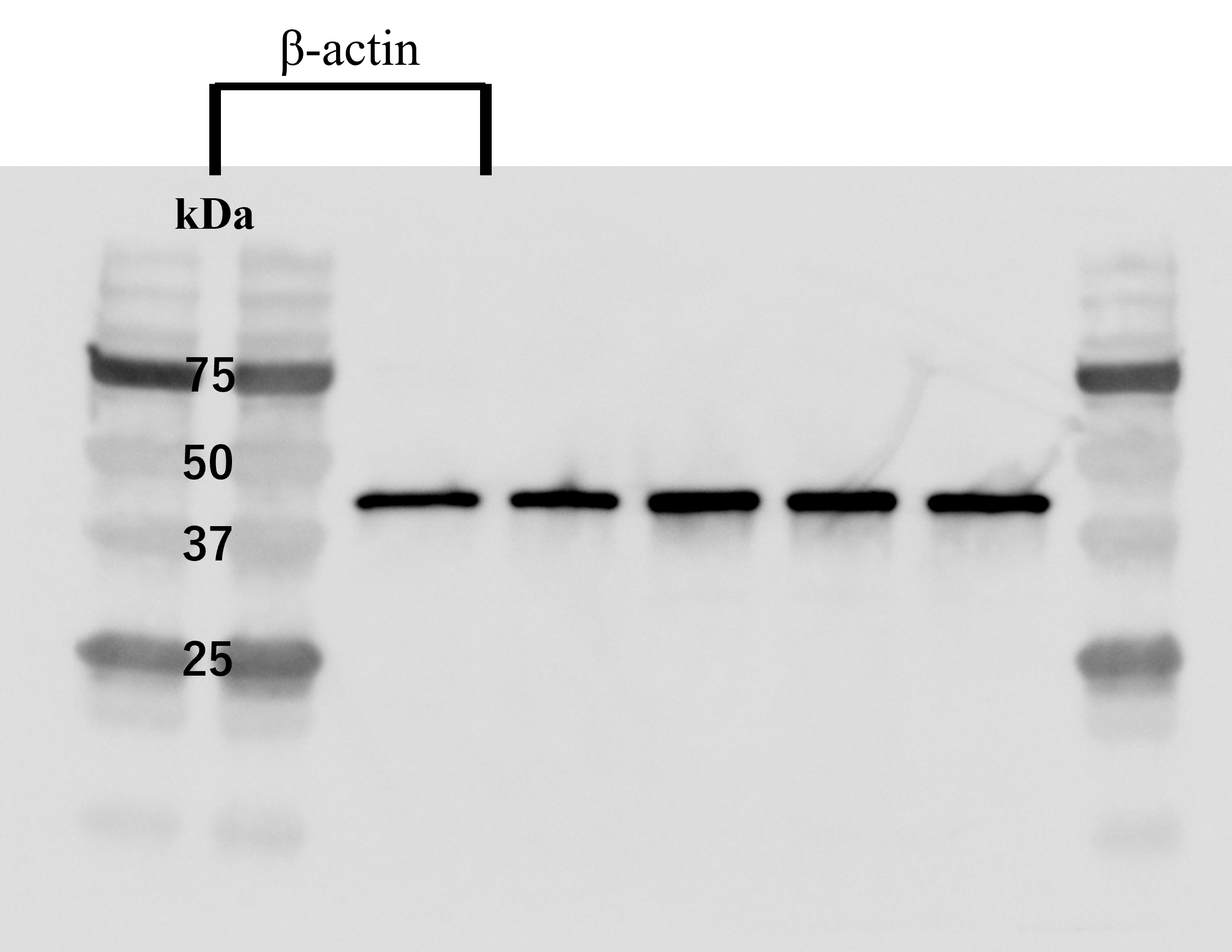

Supplement: Supplementary file 2 — Supplementary Figure 2. [file 41598_2024_52029_MOESM2_ESM.tif]

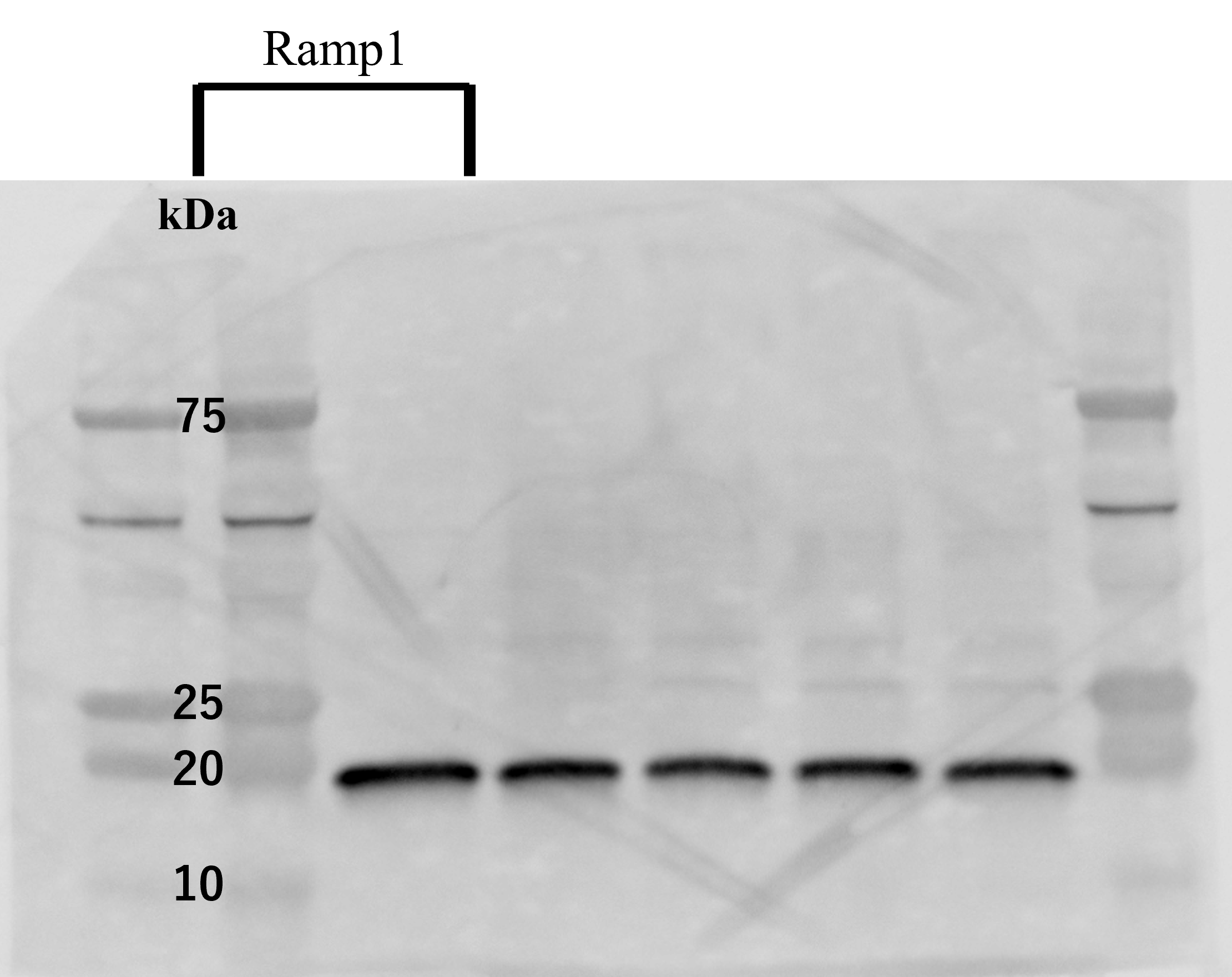

Supplement: Supplementary file 3 — Supplementary Figure 3. [file 41598_2024_52029_MOESM3_ESM.tif]
